# Contextual Flexibility in Pseudomonas aeruginosa Central Carbon Metabolism during Growth in Single Carbon Sources

**DOI:** 10.1128/mBio.02684-19

**Published:** 2020-03-17

**Authors:** Stephen K. Dolan, Michael Kohlstedt, Stephen Trigg, Pedro Vallejo Ramirez, Clemens F. Kaminski, Christoph Wittmann, Martin Welch

**Affiliations:** a Department of Biochemistry, University of Cambridge, Cambridge, United Kingdom; b Institute for Systems Biotechnology, Saarland University, Saarbrücken, Germany; c Department of Chemical Engineering, University of Cambridge, Cambridge, United Kingdom; Centro de Investigaciones Biológicas; California Institute of Technology

**Keywords:** *Pseudomonas aeruginosa*, carbon metabolism, denitrification, proteomics, carbon flux, acetate metabolism, glycerol metabolism

## Abstract

Pseudomonas aeruginosa is an opportunistic human pathogen, particularly noted for causing infections in the lungs of people with cystic fibrosis (CF). Previous studies have shown that the gene expression profile of P. aeruginosa appears to converge toward a common metabolic program as the organism adapts to the CF airway environment. However, we still have only a limited understanding of how these transcriptional changes impact metabolic flux at the systems level. To address this, we analyzed the transcriptome, proteome, and fluxome of P. aeruginosa grown on glycerol or acetate. These carbon sources were chosen because they are the primary breakdown products of an airway surfactant, phosphatidylcholine, which is known to be a major carbon source for P. aeruginosa in CF airways. We show that the fluxes of carbon throughout central metabolism are radically different among carbon sources. For example, the newly recognized “EDEMP cycle” (which incorporates elements of the Entner-Doudoroff [ED] pathway, the Embden-Meyerhof-Parnas [EMP] pathway, and the pentose phosphate [PP] pathway) plays an important role in supplying NADPH during growth on glycerol. In contrast, the EDEMP cycle is attenuated during growth on acetate, and instead, NADPH is primarily supplied by the reaction catalyzed by isocitrate dehydrogenase(s). Perhaps more importantly, our proteomic and transcriptomic analyses revealed a global remodeling of gene expression during growth on the different carbon sources, with unanticipated impacts on aerobic denitrification, electron transport chain architecture, and the redox economy of the cell. Collectively, these data highlight the remarkable metabolic plasticity of P. aeruginosa; that plasticity allows the organism to seamlessly segue between different carbon sources, maximizing the energetic yield from each.

## Abstract (precis)

Pseudomonas aeruginosa is an opportunistic human pathogen that is well known for causing infections in the airways of people with cystic fibrosis. Although it is clear that P. aeruginosa is metabolically well adapted to life in the CF lung, little is currently known about how the organism metabolizes the nutrients available in the airways. In this work, we used a combination of gene expression and isotope tracer (“fluxomic”) analyses to find out exactly where the input carbon goes during growth on two CF-relevant carbon sources, acetate and glycerol (derived from the breakdown of lung surfactant). We found that carbon is routed (“fluxed”) through very different pathways during growth on these substrates and that this is accompanied by an unexpected remodeling of the cell’s electron transfer pathways. Having access to this “blueprint” is important because the metabolism of P. aeruginosa is increasingly being recognized as a target for the development of much-needed antimicrobial agents.

## INTRODUCTION

Pseudomonas aeruginosa is an opportunistic pathogen. This cosmopolitan microbe has become one of the most frequently detected causative agents of nosocomial infection (1). It is also well known for colonizing the airways of cystic fibrosis (CF) patients. Those airways represent a spatially and chemically heterogenous environment characterized by gradients of oxygen and nutrients. To survive in this niche, P. aeruginosa must therefore overcome numerous challenges (2, 3). Indeed, recent studies have suggested that CF-adapted P. aeruginosa exhibits distinct physiological adaptations, including a tailored preference for specific carbon sources, increased requirement for oxygen, and decreased fermentation (4, 5). Moreover, and despite extensive studies into the physiology and metabolism of P. aeruginosa, we still lack a clear understanding of how the growth and assimilation of carbon is controlled in this organism. Uncovering these processes is central to developing future treatment strategies.

Carbon source utilization by P. aeruginosa is hierarchical, and the organism displays marked diauxy during growth on mixed carbon sources (6). *In vitro*, the preferred carbon sources of domesticated P. aeruginosa strains include tricarboxylic acid cycle intermediates and amino acids. Intriguingly, and unlike many enteric bacteria, glucose is not especially favored, even though it is present at millimolar concentrations in CF secretions (7). Instead, adapted CF airway isolates seem to prefer glycerol during exponential growth. The precise reason for this is unclear, as glycerol *per se* is not abundant in CF sputum (4). However, the surfactant phosphatidylcholine (PC) is abundant in CF airways, and P. aeruginosa is known to secrete lipases which can cleave PC to yield phosphorylcholine, glycerol, and long-chain fatty acids (FAs) such as palmitate. The liberated glycerol is then metabolized through the action of enzymes encoded by the *glp* operon, whereas the FAs are iteratively degraded by β-oxidation to yield acetyl CoA (acetyl-coenzyme A). The acetyl moiety is then shuttled into the tricarboxylic acid (TCA) cycle and the glyoxylate shunt to generate energy and gluconeogenic precursors for biomass production, respectively. Other sources of short-chain fatty acids in CF airways have also been recently identified (8). Indeed, acetate derived from tracheobronchial mucin breakdown by anaerobes has been reported at concentrations in excess of 5 mM in CF sputum, and reconstitution of CF airway microbiota in mucin-containing medium *in vitro* leads to >30 mM acetate accumulating (8). Glycerol metabolism and acetate metabolism therefore occupy an important crossroads in P. aeruginosa pathophysiology (9). Glycerol is also a preferred carbon source for alginate synthesis by CF isolates of P. aeruginosa and promotes the appearance of mucoid variants when present at high concentrations (10).

Despite the obvious importance of these substrates for pathophysiology, we still do not know how glycerol and acetate impact the metabolism, redox balance, and gene expression profile of P. aeruginosa. Much of what we think we know has been gleaned by extrapolation from other bacterial species, and yet those species often occupy niches very different from those occupied by P. aeruginosa and display different substrate preferences. To redress this, in the current work, we used a combination of ‘omics approaches (transcriptomics, proteomics, and ^13^C fluxomics), coupled with reverse genetics, to systematically investigate the pathway(s) of carbon assimilation during growth on acetate and glycerol. Surprisingly, the presence of these different carbon sources not only leads to a “rewiring” of central metabolism, it also gives rise to profound changes in the levels of expression of pathogenicity-associated functions, including denitrification, redox balance mechanisms, and the electron transport chain (ETC). These data underline the striking metabolic flexibility of P. aeruginosa, which allows this organism to carry out efficient, real-time free energy conservation, a trait which is likely to aid its ability to proliferate under diverse environmental conditions.

## RESULTS

### Comparative transcriptomic, proteomic, and fluxomic analysis of PAO1 cultured on glycerol or acetate as a sole carbon source reveals global changes in central carbon metabolism.

We first examined the growth characteristics of P. aeruginosa during cultivation on acetate or glycerol as a single carbon source. This revealed that P. aeruginosa grew more slowly in glycerol (μ_max_ growth rate = 0.37 ± 0.01 h^−1^) than in acetate (μ_max_ = 0.80 ± 0.01 h^−1^), glucose (μ_max_ = 0.88 ± 0.05 h^−1^), or succinate (μ_max_ = 0.87 ± 0.05 h^−1^) (see Fig. S2 in the supplemental material). To investigate this further, we examined the transcriptome, proteome, and fluxome of P. aeruginosa during the assimilation of glycerol and acetate. Our analysis was carried out on cells grown to mid-exponential phase (optical density at 600 nm [OD_600_] = 0.5) in baffled shake flasks containing MOPS (morpholinepropanesulfonic acid)-buffered media. This allowed us to elucidate specific impact of carbon source utilization on P. aeruginosa metabolism and physiology without the confounding factors of nutrient and oxygen limitation that accompany entry into the stationary phase.

**FIG 1:**
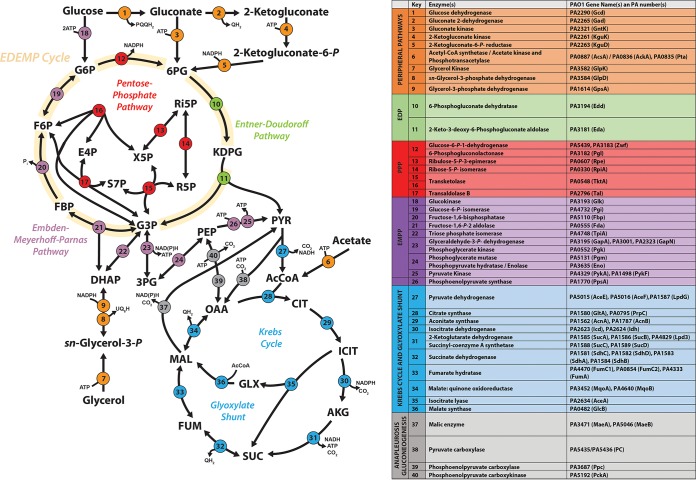
Biochemical pathways involved in central carbon catabolism in P. aeruginosa PAO1. The metabolic network was constructed around six main metabolic blocks, which are identified with different colors as follows: (i) the peripheral pathways that encompass the oxidative transformation of glucose, acetate, and glycerol (orange); (ii) the Embden-Meyerhoff-Parnas pathway (EMP; nonfunctional in P. aeruginosa due to the absence of 6-phosphofructo-1-kinase) (purple); (iii) the pentose phosphate pathway (PPP) (red); (iv) the Entner-Doudoroff pathway (EDP) (green); (v) the tricarboxylic acid cycle and glyoxylate shunt (blue); (vi) the anaplerotic and gluconeogenic bioreaction metabolic block (gray).

Acetate and glycerol have different entry points into P. aeruginosa central carbon metabolism and also are thought to have distinct effects on redox metabolism (11–14). We therefore anticipated a carbon source-specific impact on the expression of enzymes (and corresponding fluxes) involved in the relevant pathways. These pathways are summarized in Fig. 1.

Transcriptome sequencing (RNA-Seq) analyses of quadruplicate biological replicates yielded quantification of the mRNA levels from 5,578 genes. After normalization and statistical analysis (Fig. S1A to C), we identified 389 genes that displayed increased expression on acetate and 364 genes that displayed increased expression on glycerol (*P* value ≤ 0.01; log_2_ fold change greater than or equal to 2 or less than or equal to −2) (see Data Set S1 in the supplemental material). Selected examples of these modulated genes were verified using promoter-luciferase transcriptional fusions (Fig. S3) (15).

10.1128/mBio.02684-19.1 FIG S1 Transcriptomic analysis (CummeRbund) and proteomic analysis (LIMMA Package) of MOPS-acetate-grown versus MOPS-glycerol-grown grown P. aeruginosa. (A) Principal-component analysis plot (PCAplot) of RNA-Seq data. (B) Scatterplot matrix of gene-level and isoform-level expression (csScatterMatrix). (C) Scatterplot comparing the FPKM values determined under the two conditions (csScatter). (D) Principal-component analysis of proteomic data. (E) Heat map and hierarchical clustering of proteomic data. (F) Pairwise comparisons of proteomic data. Download FIG S1, TIF file, 2.4 MB. Copyright © 2020 Dolan et al. 2020 Dolan et al. This content is distributed under the terms of the Creative Commons Attribution 4.0 International license.

10.1128/mBio.02684-19.8 DATA SET S1 Summary of the electron transport chain-associated omic changes described in the main text. Full transcriptomic and proteomic data are provided in Table B and Table C, respectively. Download Data Set S1, XLSX file, 1 MB. Copyright © 2020 Dolan et al. 2020 Dolan et al. This content is distributed under the terms of the Creative Commons Attribution 4.0 International license.

**FIG 2:**
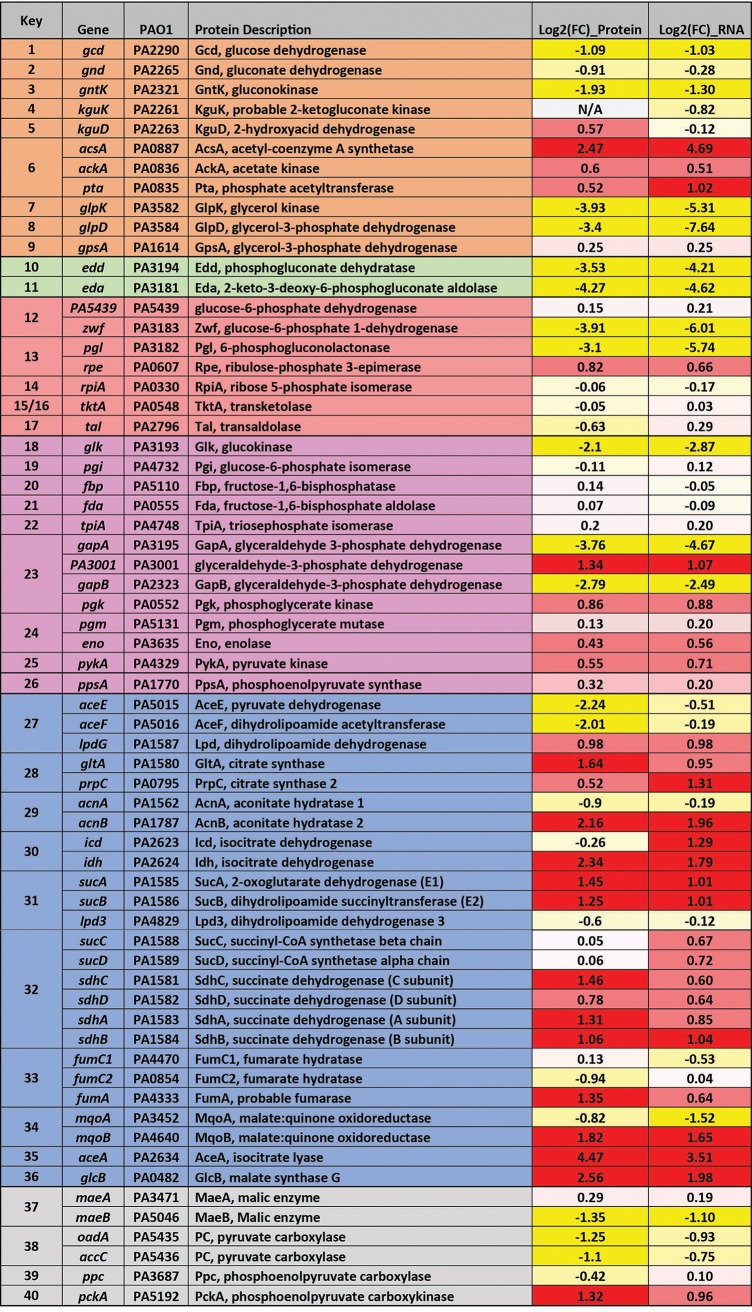
Comparison between protein and transcript fold changes (FC) for selected P. aeruginosa enzymes involved in central carbon metabolism. The figure shows the log_2_ fold changes in protein and transcript levels in (i) the peripheral pathways that encompass the oxidative transformation of glucose, acetate, and glycerol and the corresponding phosphorylated derivatives of these metabolites (orange); (ii) EMP pathway (nonfunctional, due to the absence of 6-phosphofructo-1-kinase activity) (purple); (iii) the pentose phosphate (PP) pathway (red); (iv) the upper ED pathway (green); (v) the tricarboxylic acid cycle and glyoxylate shunt (blue); and (vi) anaplerotic and gluconeogenic bioreactions (gray). RNA-Seq and proteomic data are shown in Data Set S1. Correlation plots are shown in Data Set S3.

A complementary proteomic analysis reproducibly detected a total of 3,921 proteins across three replicates for each condition. To our knowledge, this is the most comprehensive (in terms of coverage) transcriptome and proteome analysis carried out on P. aeruginosa to date. Following normalization and statistical analysis (Fig. S1), we identified 429 proteins that showed increased abundance during growth on acetate and 402 proteins that displayed increased expression on glycerol (*P* value ≤ 0.01; log_2_ fold change greater than or equal to 2 or less than or equal to −2) (Data Set S1). There was a clear relationship between the transcriptome fold changes and proteome fold changes, particularly with respect to central carbon metabolism (Fig. 2; see also Data Set S3).

**FIG 3:**
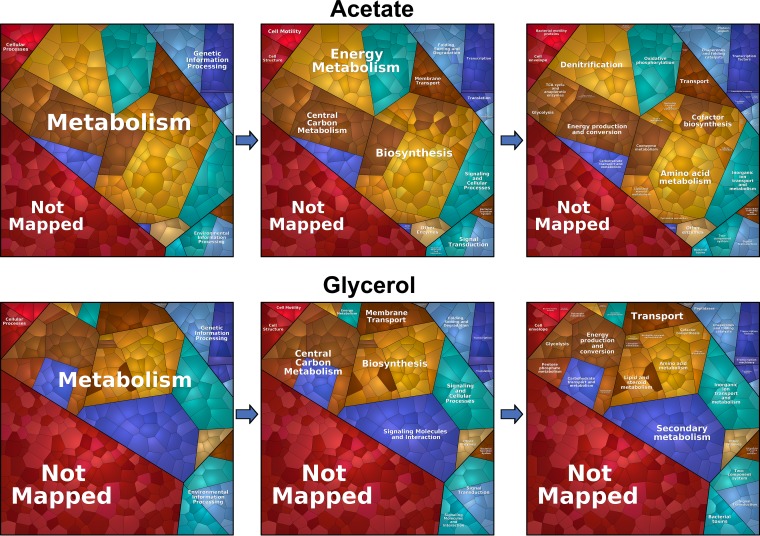
Growth on different carbon sources primarily affects metabolism. The statistically significant changes (*P* value of ≤ 0.01; fold change value of greater or equal to 1 or less than or equal to −1) during growth on glycerol and acetate are illustrated as Voronoi tree maps, which were created using the proteomaps Web service (15). Most of the proteomic changes were centered on “metabolism,” notably, “central carbon metabolism,” ‘biosynthesis,” “signaling and cellular process,” and “energy metabolism.” Pathway assignment was performed using the Kyoto Encyclopedia of Genes and Genomes (KEGG) data set. Proteome alterations which could not be assigned to a specific pathway (uncharacterized/hypothetical proteins) are indicated as “Not Mapped.”

To enable a more detailed analysis of the ‘omic alterations between these conditions, we used the proteomaps Web service (16) to illustrate the statistically significant changes (*P* value ≤ 0.01; log_2_ fold change greater than or equal to 2 or less than or equal to −2) as Voronoi tree maps. This provided a global overview of the proteomic consequences of growth in each carbon source. As shown in Fig. 3, most of the proteomic changes were centered on “central carbon metabolism,” “biosynthesis,” “signaling and cellular process,” and “energy metabolism.”

**FIG 4:**
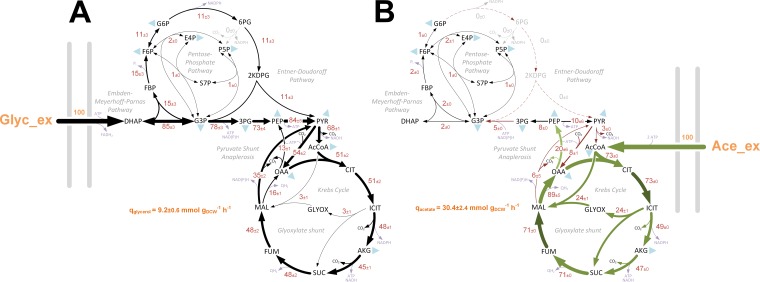
*In vivo* carbon flux distributions in central metabolism of P. aeruginosa PAO1 during growth on glycerol (A) or acetate (B) as the sole carbon source. Flux is expressed as a molar percentage of the average glycerol (9.2 mmol g^−1^ h^−1^) or acetate (30.4 mmol g^−1^ h^−1^) uptake rate, calculated from the individual rates in Data Set S2. Anabolic pathways from 11 precursors to biomass are indicated as filled blue triangles. The flux distributions with bidirectional resolution (i.e., net and exchange fluxes), including the drain from metabolic intermediates to biomass and confidence intervals of the flux estimates, are provided in Data Set S2. In agreement with previous studies of flux in P. aeruginosa and Pseudomonas fluorescens (20, 22, 23), we found no evidence for significant metabolite export during exponential growth in minimal media. The errors given for each flux reflect the corresponding 90% confidence intervals. The full flux data sets are presented in Data Set S2. Colors qualitatively indicate fluxomic correlation with changes on the protein/transcript level during growth in acetate (light green or red arrows indicate significant upregulation or downregulation, respectively; dark green or red arrows less-significant upregulation or downregulation, respectively.

Several recent studies have analyzed the metabolic interactions of clinically relevant bacteria within their host environment (17, 18) and metabolic differences between mutant strains (19). However, published fluxome studies for *Pseudomonas* species are scarce. In addition, all P. aeruginosa metabolic flux analysis (MFA) studies reported to date have been carried out using glucose as a sole carbon source (20–23), and yet this substrate is not thought to play a major role during CF airway colonization (7, 24, 25). To provide insight into the absolute metabolic fluxes of P. aeruginosa during growth on acetate and glycerol, we carried out a ^13^C fluxome analysis. This was done by measuring the mass isotopomer distributions of proteinogenic amino acids and cell carbohydrates (glycogen, glucosamine) using three separate tracers per carbon source (see Materials and Methods) (26). The calculated relative fluxes for the wild-type strain cultured on labeled glycerol or acetate are shown in Fig. 4. Comparison of the flux maps, in combination with the proteomic/transcriptomic data, generated unparalleled insights into the central carbon metabolic network(s) of P. aeruginosa.

10.1128/mBio.02684-19.9 DATA SET S2 ^13^C fluxomics data of MOPS-acetate-grown and MOPS-glycerol-grown P. aeruginosa, including calculations for anabolic demand (A), OpenFLUX SimVector files (B), reaction network (C), goodness of fit (D), and metabolic fluxes (E). Download Data Set S2, XLSX file, 0.1 MB. Copyright © 2020 Dolan et al. 2020 Dolan et al. This content is distributed under the terms of the Creative Commons Attribution 4.0 International license.

### Glycerol metabolism.

During growth on glycerol, there was strong induction of the glycerol uptake system at both the proteomic and transcriptomic levels (Fig. 2; see also Data Set S1). Glycerol is assimilated through the processes of uptake (GlpF), phosphorylation (GlpK), and dehydrogenation (GlpD) to yield dihydroxyacetone phosphate (DHAP) (27–29). Commensurate with this, expression of all three enzymes/transporters (and of their corresponding transcripts) was strongly stimulated during growth on glycerol.

Once synthesized, DHAP has two possible fates. One is anabolic. Here, DHAP is isomerized to generate glyceraldehyde 3-phosphate (G3P) through the action of triose phosphate isomerase (TpiA). DHAP and G3P are subsequently converted into fructose 1,6-*bis*-phosphate (F1,6BP), then to fructose 6-phosphate (F6P), and finally to glucose 6-phosphate (G6P) through the action of Fda, Fbp, and Pgi, respectively. This route is a necessary prerequisite for the generation of hexose sugars for cell wall synthesis and biomass production. The alternative fate of DHAP is catabolic. Here, G3P is converted to 1,3-*bis*-phosphoglycerate for glycolytic energy production (30). However, recent analyses of carbon fluxes in Pseudomonas putida suggested that the seemingly distinct anabolic and catabolic fates of DHAP/G3P may be more closely intertwined than previously thought and that a proportion of the triose phosphate carbon skeletons are recycled rather than continuing to pyruvate via the lower glycolytic reactions (31). This “EDEMP cycle” incorporates elements of the Entner-Doudoroff (ED) pathway, the Embden-Meyerhof-Parnas (EMP) pathway, and the pentose phosphate (PP) pathway. In glucose-grown P. aeruginosa and P. putida, the EDEMP cycle operates in a markedly asymmetrical manner, with most (ca. 90%) of the flux proceeding through the ED pathway-catalyzed reactions as follows: glucose → gluconate → 6-phosphogluconate → 2-keto-3-deoxy-6-phosphogluconate → G3P/pyruvate (26). Intriguingly, and in spite of the anticipated demand for hexose synthesis, our data indicate that this asymmetry is retained during growth on glycerol, since the enzymes catalyzing the “catabolic” reactions (Zwf, Pgl, Edd, and Eda) are strongly upregulated, whereas the enzymes catalyzing the “anabolic” reactions (TpiA, Fda, Fbp, and Pgi) remain relatively unaffected. We also note that glucokinase (Glk) and gluconokinase (GntK) were upregulated during growth on glycerol. Intriguingly, this indicates that even in the absence of glucose, glycerol stimulates expression of the full ED glycolytic pathway (32).

Consistent with the proteomic/transcriptomic data, when P. aeruginosa was grown on glycerol, the fluxomics indicated that about 15% of the triose phosphates (G3P + DHAP) were diverted into the EDEMP cycle to form hexose phosphates (31). Hexose generation is necessary for the synthesis of fructose 6-phosphate (F6P) and glucose 6-phosphate (G6P), required for biomass production. This recycling may also have a secondary function, since it generates NADPH through the action of glucose 6-phosphate dehydrogenase (Zwf). NADPH is a source of reducing agent for anabolism and for dealing with oxidative insult (31). However, the majority of the glycerol-derived carbon was oxidized to pyruvate through reactions in the lower half of the ED pathway. This notwithstanding, a significant amount of pyruvate was also generated from TCA-derived malate via the action of malic enzyme, feeding the so-called “pyruvate shunt.” The pyruvate shunt (a cyclical series of reactions converting malate → pyruvate → oxaloacetate → malate) generates NADPH for anabolism at the cost of consuming 1 equivalent of ATP. P. aeruginosa lacks phosphogluconate dehydrogenase activity (a major source of NADPH in many other species), so this apparently futile cycling reaction may be of major importance for growth. Indeed, more than two-thirds of the carbon in the malate pool was shuttled back to pyruvate through the action of malic enzyme, with less than one-third being converted to oxaloacetate via the malate dehydrogenase reaction. Overall, glycerol metabolism seems to be characterized by operation of the EDEMP cycle, net catabolism of G3P to pyruvate, and cycling of the latter through the pyruvate shunt.

### Acetate metabolism.

Growth on acetate as a sole carbon source elicited a similarly informative set of changes. There was a strong induction of the glyoxylate shunt enzymes (AceA and GlcB), which are known to be essential for growth on acetate (33, 34), and of enzymes directly involved in acetate activation (such as AcsA, AckA, and Pta) and acetate uptake (P. aeruginosa 3234 [PA3234]) (35). The TCA cycle-associated enzymes were almost all upregulated during growth on acetate, as was the membrane-bound malate-quinone oxidoreductase, MqoB (Fig. 2). Unlike many bacteria, P. aeruginosa does not encode a soluble NADH-producing malate dehydrogenase, and MqoB directly donates the abstracted electrons to the membrane quinone pool (36).

Carbon fluxes in acetate-grown cultures were very different from those observed in glycerol-grown cells. First, the net flux through the lower reactions of the ED pathway was in the gluconeogenic direction. Second, the flow of carbon through the EDEMP cycle was much lower than that observed in glycerol, and the flux terminated at the gluconeogenic endpoint, G6P. The absence of flux through the G6P dehydrogenase (Zwf) reaction following this point is significant, since this reaction is often thought of as a major source of NADPH for anabolism. Third, and compounding this, the extent of carbon cycling through the NADPH-producing pyruvate shunt was low. Fourth, most of the carbon for anabolism was derived from oxaloacetate rather than malate. Presumably, this may reflect the increased rate of conversion of malate to oxaloacetate by the acetate-induced malate:quinone oxidoreductase (MqoB). However, perhaps the most notable difference was that during steady-state growth on acetate, around one-third of the carbon reaching the TCA cycle-glyoxylate shunt branch point was redirected into the glyoxylate shunt. (In glycerol-grown cells, only around 3% of the carbon reaching this branch point was redirected to the glyoxylate shunt.) The glyoxylate shunt serves to supply the cell with malate and thence (following the MqoB-catalyzed conversion) also the gluconeogenic precursor, oxaloacetate. In an elegant feedback loop, high levels of oxaloacetate (such as would accumulate if the NADPH supply for anabolism was limiting) stimulate the activity of one of the isocitrate dehydrogenase isozymes, IDH, thereby restoring flux through the TCA cycle (37). One outcome of this is that NADPH levels become replenished because the IDH-catalyzed reaction is a major source of this coenzyme *in vivo*. This presumably relieves the limitation on oxaloacetate usage in anabolism. At the steady state, our flux data show that around two-thirds of the carbon flowing through the branch point is fluxed through the isocitrate dehydrogenases.

Our flux data show that no carbon passes through the NADPH-generating steps of the ED pathway during growth on acetate. So far, we have primarily framed our assessment of these observations around the need for NADPH in anabolism. However, it has not escaped our attention that NADPH limitation may impact on infection too. Several earlier workers have suggested that flux through the ED pathway may confer an additional benefit to P. aeruginosa by providing sufficient reducing power (with respect to NADPH) to counteract host-mediated oxidative stress (22, 38). Whereas our data neither confirm nor refute this notion, we show here that the following four major NADPH-supplying reactions are accessible, depending on the substrate: transhydrogenation (NADH → NADPH), the EDEMP cycle reaction, the pyruvate shunt (malic enzyme) reaction, and the isocitrate dehydrogenase(s)-catalyzed reaction.

### Beyond central metabolism—growth in acetate induces extensive remodeling of the electron transport chain.

Aerobic respiration reoxidizes NADH, thereby generating energy, maintaining redox homeostasis, and ensuring continued oxidative metabolism (39, 40). Bacteria are known to coordinate the composition of the electron transport chain (ETC) and, in particular, the levels of the terminal oxidases, according to their metabolic needs (41). The ETC of P. aeruginosa contains three NADH dehydrogenases, namely, the multisubunit proton pumping NDH-1 (encoded by *nuoA* to *nuoN* [PA2637 to PA2649]), the HQNO (*N*-oxo-2-heptyl-4-hydroxyquinoline)-resistant proton pump Nqr (encoded by *nqrA* to *nqrF* [PA2994 to PA2999]), and a single-subunit flavoenzyme which does not participate in ion translocation (NDH-2, encoded by *ndh* [PA4538]) (42). All three dehydrogenases displayed increased expression during growth on acetate, particularly at the protein level (Data Set S1).

A characteristic feature of the P. aeruginosa respiratory chain is its use of high-affinity terminal oxidases, even during aerobic growth. This unusual wiring of the P. aeruginosa ETC is thought to drive the formation of a microaerobic environment, a trait that may give the pathogen a fitness advantage during infection (43). Expression of the terminal oxidases in P. aeruginosa is directly or indirectly controlled by a two-component system, RoxS-RoxR. The kinase, RoxS, is thought to sense the redox status of the respiratory chain either by titrating the redox status of the ubiquinone/ubiquinol (UQ) pool or by responding to electron flux through the terminal oxidases. UQ acts as the electron donor for complex III, the quinol oxidases (Cyo), and the cyanide-insensitive terminal oxidase, CIO. Complex III transfers electrons to a *c*-type cytochrome, which then acts as the electron donor for the terminal cytochrome oxidases Cox, Cco1, and Cco2. Cco1 is constitutively expressed at high levels, whereas Cco2 is thought to support growth under conditions of low-oxygen tension (∼2% O_2_), as it is regulated by the anaerobic sensor Anr (44). The ‘omics data indicated that Cco1, Cco2, and Cyo and complex III subunits (encoded by PA4429 to PA4431) were highly expressed during growth on acetate, whereas the Cox oxidase was more highly expressed during growth on glycerol. This may be a strategy to limit oxidative stress, as Cox has a high H^+^/e^−^ ratio and can extract more energy per unit of carbon (45, 46). The higher level of expression of Cox during growth on glycerol may be a consequence of RoxSR-mediated inhibition of *cox* gene expression during growth on acetate (Data Set S1) (47).

Levels of the soluble pyridine nucleotide transhydrogenase Sth (PA2991) were increased ca. 3-fold during growth on acetate. Pyridine nucleotide transhydrogenases catalyze the reversible reduction of either NAD^+^ or NADP^+^ by NADPH or NADH, respectively. The primary physiological role of Sth is thought to be in the NAD^+^-dependent reoxidation of NADPH (48). Reoxidation of excess NADPH is likely to be important during growth on acetate, as extensive catabolism of this substrate through the TCA cycle generates more NADPH than is required for biosynthesis (49). Interestingly, the NAD(P) transhydrogenase encoded by *pntAA* and *pntAB* was not differentially expressed during growth on acetate or glycerol, suggesting that Sth is the main transhydrogenase used by P. aeruginosa under these conditions (48).

As noted earlier, P. aeruginosa cultured in glycerol has a significantly lower growth rate than cells grown in acetate, glucose, or succinate (Fig. S2). This lower growth rate may result in a lower metabolic demand, meaning that these cells do not require high expression of terminal oxidases and other ETC components. In contrast, higher growth rates may result in accumulation of NADH due to the rate limitations inherent in ETC-dependent reoxidation. This is because faster-growing cells are known to increase their length (50), suggesting that they are likely to have lower surface-to-volume ratios. This limits the membrane’s physical capacity for accommodating respiratory complexes and thereby also limits NADH reoxidation (51). To investigate this possibility further, we examined the length of exponentially growing P. aeruginosa cells grown in MOPS (morpholinepropanesulfonic acid) media with various carbon sources. To do this, we introduced an enhanced green fluorescent protein (eGFP)-expressing plasmid (pMF230) to PAO1 and cultured the strain in MOPS medium containing different carbon sources (52). When cells reached an OD_600_ of 0.5, they were fixed and examined by fluorescence microscopy. As shown in Fig. S6 (see also Data Set S3), the slower-growing cells in MOPS-glycerol were indeed significantly shorter than the cells grown in acetate, glucose, or succinate (50). This supports the notion that P. aeruginosa cell size (and hence, surface-to-volume ratio) is dependent on growth rate.

10.1128/mBio.02684-19.2 FIG S2 NADH/NAD^+^ ratios alongside CFU data from cells grown in MOPS. (A) Acetate. (B) Glycerol. (C) Glucose. (D) Succinate. NADPH/NADP^+^ ratios alongside CFU data from cells grown in MOPS. (E) Acetate. (F) Glycerol. (G) Glucose. (H) Succinate. Values represent total NADP(H) concentrations detected over multiple time points from cells grown in MOPS. (I) Acetate. (J) Glycerol. (K) Glucose. (L) Succinate. Values represent total NAD(H) concentrations detected over multiple time points from cells grown in MOPS. (M) Acetate. (N) Glycerol. (O) Glucose. (P) Succinate. Three biological replicates were analyzed per time point. Download FIG S2, TIF file, 1.9 MB. Copyright © 2020 Dolan et al. 2020 Dolan et al. This content is distributed under the terms of the Creative Commons Attribution 4.0 International license.

10.1128/mBio.02684-19.3 FIG S3 Luciferase detection in wild-type P. aeruginosa PAO1 carrying chromosomal promoter. Data represent *lux* fusions for the promoter regions indicated above for cells grown on various carbon sources, including MOPS-acetate, MOPS-glucose, MOPS-glycerol, and MOPS-succinate. (A) *aceA*. (B) *glcB*. (C) *cco1*. (D) *cco2*. (E) *cox*. (F) *cyo*. (G) *glpD*. (H) *nir*. Values were normalized to OD_600_ (RLU/OD_600_). Three biological replicates were analyzed per sample. Data were analyzed in GraphPad Prism (V 6.01) using *t* test statistical analysis (MOPS-glycerol versus MOPS-acetate, MOPS-glucose, or MOPS-succinate). Download FIG S3, TIF file, 1.8 MB. Copyright © 2020 Dolan et al. 2020 Dolan et al. This content is distributed under the terms of the Creative Commons Attribution 4.0 International license.

10.1128/mBio.02684-19.10 DATA SET S3 Correlation plot calculations to illustrate the log2 fold change differences in the transcriptomic, proteomic, and fluxomic data (A to C) and cell size data (D). Download Data Set S3, XLSX file, 0.1 MB. Copyright © 2020 Dolan et al. 2020 Dolan et al. This content is distributed under the terms of the Creative Commons Attribution 4.0 International license.

### Induction of the denitrification apparatus in P. aeruginosa during aerobic growth.

When O_2_ is limiting, P. aeruginosa can use nitrate as an alternative electron acceptor. This is made possible by the presence of nitrate reductase (NAR), which can accept electrons from the UQ pool, and nitrite reductase (NIR), which receives electrons via complex III and cytochrome *c* (53). Denitrification is thought to be an important pathway to support the anoxic growth of P. aeruginosa in the CF airways (54, 55), and nitrate is a known component of human body fluids, arising from diet and NO auto-oxidation. Indeed, NO_3_^−^ concentrations in the CF airways have been reported to be between 73 μM and 792 μM, with an average of 348 μM (56). Molecular oxygen is therefore not essential for growth in this environment.

The gene clusters encoding the dissimilatory nitrate reductases (*nar* and *nap*) and those encoding nitrous oxide (N_2_O) utilization (*nos*) are unlinked, whereas the genes encoding nitrite respiration (*nir*) and nitric oxide respiration (*nor*) are adjacent. In addition to the enzymes named above, the denitrification operons harbor genes for ancillary functions such as cofactor synthesis, transport, and protein maturation (53). Both the proteomic and transcriptomic analyses showed activation of the denitrification pathways following growth in MOPS-acetate (Data Set S1). This was surprising, since the cultures were harvested during exponential growth in baffled, well-aerated conical flasks without added nitrate. This is an important point, because denitrification in P. aeruginosa is known to be under the control of the master regulator Dnr. In turn, Dnr is under the control of the anaerobic transcriptional regulator Anr. The latter contains an oxygen-sensitive [4Fe-4S]^2+^ cluster and is thought to be active only under conditions of oxygen limitation. In contrast, Dnr is a nitric oxide (NO) sensor and contains a ferrous heme. Dnr protein expression was upregulated 2.8-fold during growth on acetate compared with glycerol (Data Set S1). Previous work has shown that, in addition to Dnr-mediated regulation, the *narGHJI* operon is also activated by the NarXL two-component system. The NarX and NarL proteins were upregulated 2-fold during growth on acetate. Taken together, these data indicate that in spite of the presence of molecular oxygen and the absence of added nitrate, growth on acetate strongly induces the denitrification apparatus in comparison with glycerol. We conclude that the denitrification apparatus and components required for aerobic respiration are expressed simultaneously during growth on acetate.

### P. aeruginosa cellular NAD(P)(H) ratios change in a carbon source dependent manner.

**FIG 5:**
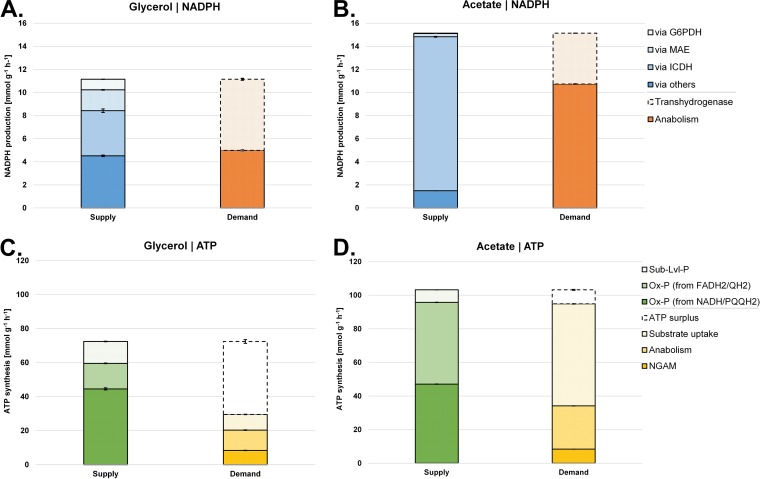
Quantitative analysis of NADPH supply and demand (redox) for glycerol-grown (A) and acetate-grown (B) P. aeruginosa. Data represent ATP (energy metabolism) supply and demand for glycerol-grown (A) and acetate-grown (B) P. aeruginosa (see Fig. 6). Values representing reactions linked to NADPH (A and B) and ATP (C and D) metabolism were calculated from the obtained fluxes (see Fig. 4). Values are given as absolute fluxes (in millimoles per gram per hour) and are related to the specific carbon uptake rate (see Data Set S2). G6PDH, glucose 6-phosphate dehydrogenase; MAE, malic enzyme; ICDH, isocitrate dehydrogenase; Ox-P, oxidative phosphorylation; NGAM, non-growth-associated maintenance needs.

**FIG 6:**
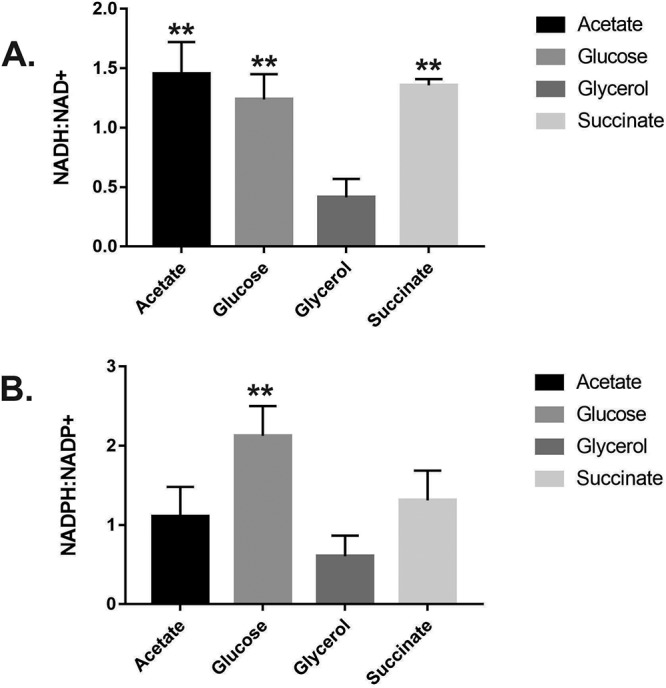
Maximal measured NADH/NAD^+^ and NADPH/NADP^+^ ratios in P. aeruginosa grown in the indicated sole carbon sources. Exponentially growing cells in MOPS-glycerol showed a significantly lower NADH/NAD^+^ ratio (*P* < 0.01) than cells grown in MOPS-acetate, MOPS-glucose, or MOPS-succinate and a significantly lower NADPH/NADP^+^ ratio than cells grown in MOPS-glucose (*P* = 0.0064). The total NAD(P)(H) concentrations seen at each time point and with each carbon source are shown in Fig. S2 (see also Fig. S4). The data were analyzed using GraphPad Prism (v 6.01) and *t* test statistical analysis (MOPS-glycerol versus MOPS-acetate, MOPS-glucose, or MOPS-succinate).

By summing the fluxes through reactions that either produce or consume NADPH or ATP, we were able to calculate the relative contributions of different metabolic reactions to the redox (NADPH) and energy (ATP) balances during growth on each carbon source (Fig. 5). This revealed that cells grown on glycerol obtain their NADPH from a variety of reactions, whereas acetate-grown cells are heavily reliant on NADPH derived from the reactions associated with isocitrate dehydrogenase(s). Moreover, most of the NADPH generated during growth on acetate was used in anabolism (Fig. 6). With regard to ATP, cells grown on glycerol were effectively “oversupplied” with this reagent, maintaining a substantial “operating surplus” of ATP. In contrast, the supply of ATP in cells grown on acetate was essentially identical to the metabolic demand. The most likely reason for this is the 2 × ATP “cost” of acetate uptake and activation (57). During growth on glycerol, the cell generates 2.6 mol of ATP per mole of C, whereas just 1.1 mol of ATP is generated on acetate. This “energy deficit” may explain why the cell induces additional mechanisms to maximize energy production during growth on acetate (e.g., high-affinity electron acceptors, increased denitrification, increased Sth transhydrogenase expression, etc. [Data Set S1]).

10.1128/mBio.02684-19.4 FIG S4 (A) CFUs. (B) NAD(H) concentrations. (C) NADP(H) concentrations. Data represent concentrations extracted from wild-type PAO1 grown in MOPS-acetate with or without 20 mM KNO_3_. Three biological replicates were analyzed per time point. (D) CFUs. (E) NAD(H) concentrations. (F) NADP(H) concentrations extracted from PAO1 wild-type (WT) and Δ*dnr* cells grown in MOPS-acetate with or without 20 mM KNO_3_. Three biological replicates were analyzed per time point. Download FIG S4, TIF file, 2.7 MB. Copyright © 2020 Dolan et al. 2020 Dolan et al. This content is distributed under the terms of the Creative Commons Attribution 4.0 International license.

**FIG 7:**
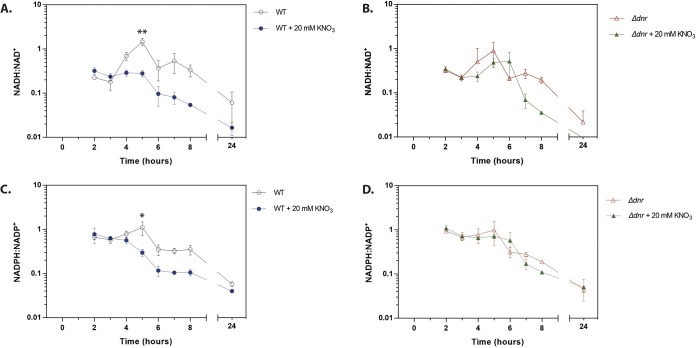
Coenzyme reoxidation is impaired in a *dnr* mutant. The NADH/NAD^+^ (A and B) and NADPH/NADP^+^ (C and D) ratios were measured in cultures of wild-type (WT) PAO1 (A and C) and in cultures of an isogenic Δ*dnr* mutant (B and D). Cultures were grown in MOPS-acetate with or without 20 mM KNO_3_, as indicated. The corresponding CFU counts are shown in Fig. S4. The data were analyzed using GraphPad Prism (v 6.01) using *t* test statistical analysis at 5 h of growth. Nitrate addition to the PAO1 wild-type strain resulted in significant reductions in the NADH/NAD^+^ ratio (**, *P* = 0.0017) and the NADPH/NADP^+^ ratio (*, *P* = 0.0202). Nitrate addition to a Δ*dnr* mutant did not significantly alter either ratio (*P* = 0.8065 and *P* = 0.1862, respectively).

We anticipated that the higher growth rate (Fig. S2) and TCA cycle driven metabolism (Fig. 2) during growth on acetate would result in an increased rate of respiration and that this might drive the NAD(P)H/NAD(P)^+^ ratio toward a more reduced state (51). This was indeed the case (Fig. S2), indicating that the carbon source alone is sufficient to alter the intracellular redox economy of P. aeruginosa. We next investigated what might be driving this increased NADH/NAD^+^ ratio during growth on acetate. Microorganisms display an elevated NADH/NAD^+^ ratio when NADH reoxidation is slowed by limitation of electron acceptors (58) or when the metabolism of certain carbon sources outpaces the capacity of the P. aeruginosa ETC to reoxidize the coenzyme. This may explain why cells grown in acetate appear to scavenge for alterative electron acceptors (such as nitrate, as indicated by the increased expression of the denitrification apparatus). To determine if aerobic denitrification might be used by P. aeruginosa to correct the reduced status of its redox pool, we therefore examined the impact of nitrate addition on the NADH/NAD^+^ ratio. As shown in Fig. 7, the addition of 20 mM nitrate to MOPS-acetate cultures decreased the NADH/NAD^+^ ratio during exponential growth. Absolute quantitation of the NADH and NAD^+^ levels (Fig. S2) confirmed that the levels of the total NAD(H) and NADP(H) pools were similar in the presence and absence of added nitrate. To confirm that this drop in the NADH/NAD^+^ ratio was due to aerobic denitrification, we generated a deletion mutant with a mutation in the master transcriptional regulator of denitrification, *dnr*. As expected, nitrate reductase (NirS) expression was abolished in the *dnr* mutant and also in an *anr* mutant (Anr controls the expression of *dnr*) (Fig. S5). Crucially, and consistent with findings from other laboratories showing that nitrate addition has little impact on the NADH/NAD^+^ ratio in mutants defective in denitrification (40), the *dnr* mutant was also defective in nitrate-dependent reoxidation of NADH and NADPH (Fig. 7). We conclude that aerobic denitrification during growth on acetate impacts the redox status of the NAD(P)H/NAD(P)^+^ pool.

10.1128/mBio.02684-19.5 FIG S5 Western blotting. (A) Expression of the Anr/Dnr-regulated denitrification enzyme NirS during exponential growth of P. aeruginosa in MOPS-glucose, MOPS-succinate, or MOPS-acetate. NirS was not expressed in a Δ*anr* or Δ*dnr* mutant. Isocitrate dehydrogenases ICD and IDH were used as loading controls. (B) Expression of NirS during exponential growth of P. aeruginosa in MOPS-acetate or in MOPS-acetate plus sodium nitrate (20 mM). Sodium nitrate (20 mM) was capable of inducing NirS expression in the WT strain and in a Δr*oxSR* mutant but not in a Δ*anr* or Δ*dnr* mutant. Three biological replicates were analyzed per sample. The Δ*anr*, Δ*dnr*, and Δ*roxR* lanes are representative of the triplicate analyses. Download FIG S5, TIF file, 2.1 MB. Copyright © 2020 Dolan et al. 2020 Dolan et al. This content is distributed under the terms of the Creative Commons Attribution 4.0 International license.

## DISCUSSION

Several earlier studies described the systems-level cellular adjustments that accompany the growth of industrially relevant model organisms such as Escherichia coli, Saccharomyces cerevisiae, Bacillus subtilis, Corynebacterium glutamicum, Basfia succiniciproducens, and P. putida in single carbon sources (59–64). However, and although the accrued metabolic models have made a valuable contribution toward our “textbook understanding” of microbial metabolism, it is clear that they can be extrapolated to other organisms, such as P. aeruginosa, only loosely (65). Surprisingly, a comparative ‘omics study has yet to be carried out for P. aeruginosa grown on carbon sources relevant to infection. Overall, carbon preference remains poorly understood for P. aeruginosa, even though it is known to have a profound impact on virulence-associated phenotypes, including toxin production, biofilm formation, and growth rate. Importantly, unravelling the versatility of P. aeruginosa metabolism has clear implications for the bacterial response to antibiotics and for persistence during chronic biofilm infections (66, 67). In this work, we rectified this by developing a high-resolution global map of P. aeruginosa metabolism during growth on two infection-relevant carbon sources, namely, acetate and glycerol.

Our data indicate a clear relationship between transcript and protein changes in P. aeruginosa for the growth regimens tested (21, 37, 68). These expression data enabled us to establish which metabolic pathways are active under each growth condition, thereby providing an experimentally supported framework for interpreting the fluxomic data. This notwithstanding, the fluxomics highlighted the possibility of the presence of additional layers of regulatory complexity involved in fine-tuning of central carbon metabolism. For example, growth on acetate results in the expression of three enzymes (the isocitrate dehydrogenases, ICD and IDH, and the isocitrate lyase, ICL), all of which compete for a shared substrate, isocitrate. These enzymes are known to exhibit catalytic and regulatory properties radically different from those of their E. coli counterparts (37). In spite of this, our fluxomic analysis revealed remarkably similar flux partitioning at the branch point between the TCA cycle and the glyoxylate shunt in the two organisms (37).

In addition to alterations in central carbon metabolism, we also noted substantial remodeling of the composition of the ETC during growth in acetate compared with glycerol. For example, we noted a substantial increase in expression of genes encoding terminal oxidases (genes *cyo*, *cco1*, and *cco2*) during growth on acetate and increased *cox* expression during growth on glycerol. This suggests that different terminal oxidases are employed under different growth conditions, allowing metabolism to be optimally adjusted for energy generation (69). Interestingly, growth on mucus has been previously shown to stimulate Cyo expression and to repress Cco2 expression (70). Our data suggest that these observations may be driven by the metabolism of one or more mucus-derived compounds.

The ETC alterations that we observed included strong aerobic induction of the denitrification machinery during growth on acetate. This was unexpected, since denitrification is usually associated with oxygen limitation and microaerobic growth. The signal which leads to this remodeling of the ETC remains to be elucidated. However, we speculate that these changes in ETC architecture and composition may represent a mechanism to restore redox balance (NADH/NAD^+^ ratio) in the cell (71). Acetate-grown cells accumulate NADH, so activating alternative mechanisms (such as the denitrification apparatus) to oxidize NADH may represent a homeostatic attempt to regain optimal cellular redox status. Consistent with this, the addition of nitrate to acetate-grown P. aeruginosa did lead to a more highly oxidized NADH/NAD^+^ ratio. Moreover, a mutant (Δ*dnr*) strain defective in denitrification was unable to maintain optimal redox homeostasis. However, growth was not affected in the Δ*dnr* mutant, perhaps suggesting that the organism has additional (possibly compensatory) mechanisms to deal with redox dysregulation (22, 72).

Aerobic denitrification has been recently suggested to function as a “bet-hedging” strategy to anticipate nitrate availability and to respond to abrupt anoxia (73, 74). The oxygen-limited microenvironment in the CF airways is thought to result in P. aeruginosa growth mediated by microaerobic respiration (75). One possibility is that hybrid respiration (a combination of aerobic respiration and denitrification) may well be a predominant mechanism of survival under these conditions (76). Genes under the control of the transcriptional regulator Anr are also abundant in P. aeruginosa RNA extracted from CF sputum (25, 77, 78).

A key fundamental question arises from this study: how does P. aeruginosa activate the denitrification system during growth under aerobic conditions? Until now, induction of the denitrification machinery has been largely attributed to the activity of Anr (and its subordinate denitrification-specific regulator, Dnr). Anr dimerization is thought to be dependent on the formation of an oxygen-liable [4Fe-4S]^2+^ reaction, which is destabilized and disassociates in the presence of oxygen, abolishing Anr activity. However, there is accruing evidence indicating that Anr may be active even in the presence of oxygen. For example, Jackson et al. have shown that choline catabolism leads to aerobic expression of the Anr regulon, suggesting that Anr activity can be sustained in the presence of oxygen. Also, Anr overexpression in well-aerated cultures was previously shown to increase the levels of *dnr* transcript 29-fold (79). Furthermore, expression of the *nir* and *nar* genes has been shown to increase in late-exponential-phase cultures of PA14 compared with stationary-phase cultures (80). This mobilization of the Anr regulon in the presence of oxygen suggests that Anr can modulate gene expression without fully intact metallocenters or dimers. This may represent a need to assist the cells during the transition to conditions of low-oxygen tension (79).

Finally, our data demonstrate that P. aeruginosa grew more rapidly on acetate than in glycerol, in terms of both specific growth rate (measured as optical density) and cell length (see Fig. S6 in the supplemental material). In order to maintain redox homeostasis under such conditions, cells often switch to “overflow metabolism,” whereby partially oxidized metabolic intermediates such as acetate are excreted (58, 81). At first glance, overflow metabolism seems wasteful, although it does allow maximization of the growth rate (82). It is tempting to speculate that aerobic denitrification in P. aeruginosa may also represent a form of overflow metabolism in which the alternate electron acceptor nitrate is utilized alongside oxygen in an effort to maintain redox homeostasis.

10.1128/mBio.02684-19.6 FIG S6 Bacterial cell length as determined by fluorescence microscopy of PAO1 carrying the pMF230 eGFP-expressing plasmid. Cells were grown in MOPS media with acetate, glucose, glycerol, or succinate as the sole carbon source. The calculated data points are provided in Data Set S3. Download FIG S6, TIF file, 1.4 MB. Copyright © 2020 Dolan et al. 2020 Dolan et al. This content is distributed under the terms of the Creative Commons Attribution 4.0 International license.

## MATERIALS AND METHODS

### Growth conditions.

Unless otherwise indicated, P. aeruginosa strain PAO1 (83) was routinely grown in lysogeny broth (LB Lennox) (Oxoid Ltd.) at 37°C with shaking (250 rpm). The strains used in this study are listed in Table S1 in the supplemental material. The overnight precultures were started from separate clonal source colonies on streaked LB agar plates. Strains were cultured in MOPS (morpholinepropanesulfonic acid) media with the relevant carbon sources (84). Cell growth was monitored as optical density by the use of a spectrophotometer at a wavelength of 600 nm (OD_600_). A previously determined conversion factor of 0.42 g cell dry weight (CDW) per OD unit was used to calculate biomass-specific rates and yields from the obtained OD_600_ values (22).

10.1128/mBio.02684-19.7 TABLE S1 (A) Oligonucleotide primers used in this study. (B) Bacterial strains and plasmids used in this study (15, 52, 83, 103). Download Table S1, DOCX file, 0.02 MB. Copyright © 2020 Dolan et al. 2020 Dolan et al. This content is distributed under the terms of the Creative Commons Attribution 4.0 International license.

### Transcriptome sequencing (RNA-Seq).

PAO1 was grown in 40 ml MOPS with acetate or glycerol as the sole carbon source (in quadruplicate) in baffled flasks (500 ml) at 37°C with shaking (250 rpm). An aliquot (5 ml) of culture was harvested from each sample at an OD_600_ of 0.5 (exponential-growth phase) and added to an equal volume of RNAlater. Samples were then sedimented in an Eppendorf 5810R centrifuge at 3,220 × *g* for 15 min (4°C), and the pellets were stored at –80°C. Total RNA was isolated as described previously (85), followed by phenol-chloroform-isoamyl alcohol extraction and ethanol precipitation. rRNA was then depleted from each sample (5 μg each) using a bacterial Ribo-Zero rRNA removal kit (Illumina). The integrity of the RNA was evaluated using an RNA 6000 Nano LabChip and an Agilent 2100 Bioanalyzer (Agilent Technologies, Germany). Eight indexed, strand-specific cDNA libraries were prepared, and samples were sequenced using an Illumina HiSeq 2000 system (GATC Biotech, Germany) with a 51-bp single-end read length.

### Read mapping and annotations.

The resulting FASTQ files were mapped to the PAO1 genome obtained from the Pseudomonas Genome Database (PDG) (http://www.pseudomonas.com/) using TopHat v.2.0.3 (37) and Bowtie v.0.12.8 (22) with an ∼97% success rate to generate SAM files. The sequence reads were adaptor clipped and quality trimmed using trimmomatic (86) with default parameters. Transcript abundance and differential gene expression were calculated with the program Cufflinks v.2.0.1 (87). Gene expression levels were normalized using the reported values representing fragments per kilobase per million (FPKM) mapped exon reads. Genes were considered to have been induced or repressed only when their log_2_ fold change value was greater than 1 or less than −1, respectively, and when their *P* value was <0.01 (see Data Set S1 in the supplemental material; see also Fig. S1 in the supplemental material).

### Quantitative proteomic analysis.

P. aeruginosa cells (OD_600_ = 0.5) (30 ml) were harvested under growth conditions identical to those reported in the transcriptomics section above. Pellets were resuspended in lysis buffer [100 mM Tris-HCl, 50 mM NaCl, 10% (vol/vol) glycerol, 1 mM Tris(2-carboxyethyl)phosphine) (TCEP), pH 7.5] with cOmplete Mini protease inhibitor cocktail (Roche). Following three rounds of sonication (3 × 10 s) on ice, supernatants were clarified by sedimentation (21,130 × *g*, 15 min, 4°C) in an Eppendorf 5424R centrifuge. Aliquots (100 μg) of each sample were reduced with TCEP, alkylated with iodoacetamide, and labeled with tandem mass tags (TMTs). TMT labeling was performed according to the protocol specified by the manufacturer (Thermo Fisher).

### LC-MS/MS.

Dried fractions from the high-pH reverse-phase separations were resuspended in 30 μl of 0.1% (vol/vol) formic acid (14 combined fractions). Liquid chromatography-tandem mass spectrometry (LC-MS/MS) experiments were performed using a Dionex Ultimate 3000 rapid separation LC (RSLC) nano-ultraperformance liquid chromatography (nanoUPLC) system (Thermo Fisher Scientific Inc., Waltham, MA, USA) and a Lumos Orbitrap mass spectrometer (Thermo Fisher Scientific Inc., Waltham, MA, USA). Peptides were loaded onto a Thermo Scientific PepMap 100 C_18_ precolumn (5-mm particle size, 100-Å pore size, 300-mm inner diameter [i.d.] by 5-mm length) from an Ultimate 3000 autosampler with 0.1% (vol/vol) formic acid for 3 min at a flow rate of 10 μl/min. Separation of peptides was performed by C_18_ reverse-phase chromatography at a flow rate of 300 nl/min using a Thermo Scientific reverse-phase nano EASY-spray column (Thermo Scientific PepMap C_18_; 2-mm particle size, 100-Å pore size, 75-mm i.d. by 50-cm length). Solvent A was water–0.1% formic acid, and solvent B was 80% (vol/vol) acetonitrile–20% water–0.1% formic acid. The linear gradient employed was 2% to 40% solvent B over 93 min (the total LC run time was 120 min, including a high-organic-wash step and column reequilibration).

The eluted peptides were sprayed into the mass spectrometer by means of an EASY-Spray source (Thermo Fisher Scientific Inc.). All *m*/*z* values representing eluting peptide ions were measured in an Orbitrap mass analyzer (set at a resolution of 120,000) and were scanned at between *m*/*z* 380 and 1,500 Da. Data-dependent MS/MS scans (top speed) were employed to automatically isolate and fragment precursor ions by collision-induced dissociation (CID; normalized collision energy [NCE] value, 35%) analyzed in the linear ion trap. Singly charged ions and ions with unassigned charge states were excluded from selection for MS/MS, and a dynamic exclusion window of 70 s was employed. The top 10 most abundant fragment ions from each MS/MS event were then selected for a further stage of fragmentation by synchronous precursor selection (SPS) and MS/MS/MS (MS3) (88) in the high-energy-collision cell using HCD (high-energy collisional dissociation; NCE value of 65%). The *m*/*z* values and relative abundances of each reporter ion and of all fragments (mass range, 100 to 500 Da) in each MS3 step were measured in the Orbitrap analyzer, which was set at a resolution of 60,000. This was performed in cycles of 10 MS3 events, after which the Lumos instrument reverted to scanning the *m*/*z* ratios of the intact peptide ions and the cycle continued.

### Proteomic data analysis.

Proteome Discoverer v2.1 (Thermo Fisher Scientific) and Mascot (Matrix Science) v2.6 were used to process raw data files. Data were aligned with UniProt Pseudomonas aeruginosa data (common repository of adventitious proteins [cRAP] v1.0) (5,584 sequences).

The R package MSnbase (89) was used for processing proteomics data. Protein differential abundances were evaluated using the Limma package (90). Differences in protein abundances were statistically determined using Student’s *t* test with variances moderated by the use of Limma’s empirical Bayes method. *P* value*s* were adjusted for multiple testing by the Benjamini Hochberg method (91). Proteins were considered to have increased or decreased in abundance only when their log_2_ fold change value was greater than 1 or less than −1, respectively, and when their *P* value was <0.01.

### Construction of luciferase reporter strains.

Translational reporter constructs were made by fusing the upstream promoter sequences with *luxCDABE* using the primers listed in Table S1. Purified PCR products were digested and directionally ligated into the multiple-cloning site of the pUC18T-mini-Tn7T-lux-Gm plasmid (15). The mini-Tn*7*-lux elements were integrated into the PAO1 chromosome by electroporation along with the helper plasmid pTNS2 as previously described (92).

### Luciferase promoter assay.

Luciferase and OD_600_ readings were measured using a BMG Labtech FLUOstar Omega microplate reader. Strains were cultured in MOPS media with the relevant carbon sources (100 μl) in 96-well microplates (Greiner Bio-One) (F-Bottom, black), covered with gas-permeable imaging seals (4titude; catalog no. 4ti-0516/96). Luciferase expression was assessed every 30 min (gain = 3,600) for up to 24 h. Growth was assessed by taking OD readings at 600 nm simultaneously with the luminescence readings. Luciferase readings were expressed as relative luminometer units (RLU) normalized to OD_600_ in order to control for growth rate differences across the selected carbon sources.

### ^13^C fluxomics.

Starter cultures were grown In LB medium. For the second and main cultures, PAO1 was grown in MOPS minimal media with 60 mM acetate or 40 mM glycerol as the sole carbon source (120 mM carbon). For ^13^C flux experiments, naturally labeled acetate and glycerol were replaced with three separate tracers per carbon source to maximize data set resolution and to accurately determine substrate uptake. Naturally labeled glycerol was replaced with [1,3-^13^C_2_]glycerol (99%), [2-^13^C]glycerol (99%), and an equimolar mixture of [U-^13^C_3_] glycerol (99%) (Cambridge Isotope Laboratories, Inc., Andover, MA, USA) and naturally labeled glycerol. Naturally labeled acetate was replaced with 99% [1-^13^C]sodium acetate or [2-^13^C]sodium acetate or a molar 1:1 mixture of [U-^13^C_2_] sodium acetate (Sigma-Aldrich [Poole, Dorset, United Kingdom]) and natural sodium acetate.

Starter cultures were prepared by inoculating LB medium with a loop of freshly plated PAO1. After 6 h of incubation, 50 μl of cell suspension was transferred to a second culture of MOPS minimal medium. Subsequently, exponentially growing cells were used as inoculum for main cultures, and PAO1 was cultured in 25 ml of minimal medium in 250-ml baffled shake flasks (200 rpm, 37°C) in an orbital shaker (Aquatron; Infors AG, Switzerland). These growth conditions were selected to ensure sufficient aeration during cultivation. The oxygen level was maintained above 80% of saturation under growth conditions identical to those used previously for P. aeruginosa PAO1 (22). In cultures incubated with ^13^C-labeled tracer, the inoculum level was always kept below 1% (initial OD of <0.02) of the final sampled cell concentration to exclude potential interference of nonlabeled inoculum with subsequent calculations of flux (93).

Mass isotopomer labeling analysis of proteinogenic amino acids, mass isotopomer labeling analysis of cell sugar monomers (glucose and glucosamine), and metabolic reaction network and flux calculations were carried out as described previously (26).

### Quantification of substrates and products.

Acetate and glycerol, as well as organic acids (citric acid, α-ketoglutaric acid, gluconic acid, 2-ketogluconic acid, pyruvic acid, succinic acid, lactic acid, formic acid, and fumaric acid), were quantified in filtered culture supernatants (Costar Spin-X; 0.22-μm-pore-size filters) using isocratic high-performance liquid chromatography (Agilent 1260 Infinity series; Aminex HPX-87H column) (65°C; flow rate of 0.5 ml min^−1^) equipped with refractive index (RI) and UV detectors (210 nm) with 50 mM H_2_SO_4_ as the eluent (94). Concentrations were determined from commercial standards which were analyzed on the same run. These data were then used to calculate specific uptake and formation rates and yields for acetate, glycerol, and secreted by-products (see Data Set S2 in the supplemental material).

### Calculation of redox cofactor and ATP balances.

Total production of reduced cofactors was determined by summing all cofactor-forming fluxes while considering substrate-dependent cofactor specificities (31, 36, 95). Anabolic NADPH requirements and anabolically produced NADH levels were estimated from the biomass composition (22, 96) and the measured rates of specific growth. Surplus NADPH was considered to have been converted into NADH via the activities of soluble (SthA [PA2991]) and membrane-bound, proton-translocating (PntAB [PA0195 and PA0196]) pyridine nucleotide transhydrogenases (48).

### ATP.

The total ATP demand was calculated by summing the anabolic demands of biomass building block synthesis and polymerization estimated from cell composition multiplied by the corresponding specific rate of growth on each substrate (22, 26) and additionally considering non-growth-associated maintenance (NGAM) costs (96) and ATP costs for substrate uptake (see reaction network in Data Set S2 [57]). Acetate uptake was considered a consequence of acetyl-CoA synthase (ACS) consuming 2 mol of ATP per mol of acetate catabolized (57).

The amount of ATP synthesized via oxidative phosphorylation in the respiratory chain was estimated by assuming P/O ratios of 1.875 for NADH and PQQH_2_ (97) and 1.0 for FADH_2_ and other quinone carriers (98).

The anabolic ATP requirement was calculated from published biomass composition data for pseudomonads, mainly from data representing synthesis of proteins, RNA, and lipids and their polymerization (22, 99). The NGAM for pseudomonads was previously modeled using genome-scale models (96, 99). Here, ATP surplus represents the amount of ATP available to fulfil the requirements associated with growth-associated maintenance and other cellular ATP-consuming tasks.

### NAD(P)(H) extraction.

P. aeruginosa PAO1 cultures were grown in MOPS media containing a single carbon source (40 mM acetate, 15 mM glucose, 30 mM glycerol, or 30 mM succinate) at 37°C with shaking at 250 rpm, using a culture volume of 150 ml in a 2-liter baffled Erlenmeyer flask. For each NAD(P)(H) extraction, 1.8 ml of culture was removed and immediately added to 7.5 ml ice-cold 100% methanol followed by centrifugation at 3,220 × *g* for 14 min at 4°C to obtain a cell pellet. The pellet was resuspended in 0.2 M HCl for NAD(P)^+^ or 0.2 M NaOH for NAD(P)H extraction, after which incubation was performed at 52.5°C for 10 min followed by incubation on ice for 5 min. HCl or NaOH was then neutralized by the dropwise addition of 0.1 M NaOH or 0.1 M HCl, respectively, while vortex mixing was performed at low speed. The mixture was then centrifuged for 5 min at 15,800 × *g*, and 135 μl of the supernatant was removed for immediate NAD(P)(H) measurement or storage at —80°C. Samples were stored for a maximum of 1 week before measurement.

### NAD(P)(H) measurement.

NAD(P)(H) concentrations were measured using an enzyme cycling assay in a 96-well microtiter plate (Thermo Scientific catalog no. 167008) as described previously (100). Reagent master mix containing 2 volumes 1 M bicine (pH 8.0), 1 volume 100% ethanol, 1 volume 40 mM EDTA (pH 8.0), 1 volume 4.2 mM thiazolyl blue, 2 volumes 16 mM phenazine ethosulfate, and 1 volume distilled water (dH_2_O) was prepared. The reagent mix was incubated at 30°C and primed to injectors in a BMG Labtech FLUOstar Omega microplate reader. Aliquots (15 μl) of NAD(P)(H) extracts were added to individual wells of a 96-well microtiter plate, which was then incubated in the microplate reader at 30°C. Reagent master mix (80 μl) was added via a microplate reader injector (300 μl s^−1^) and vigorously mixed (200 rpm, 3 s) followed by static incubation for 10 min. Immediately before measurement, a solution of alcohol dehydrogenase (1 mg ml^−1^ in 0.1 M bicine) was prepared for NAD(H) measurement or glucose 6-phosphate dehydrogenase (0.1 mg ml^−1^ in 0.1 M bicine) for NAD(P)(H) measurement and primed to a second injector. To start the reaction, each well was injected (300 μl s^−1^) with 5 μl of enzyme solution, followed by vigorous mixing (200 rpm, 1 s). The absorbance at 570 nm was then recorded every 30 to 60 s for 20 min, with vigorous shaking (200 rpm, 1 s) before each read. Slopes from plots representing absorbance over time were calculated for NAD(P)H and NAD(P)^+^ and were then used to calculate ratios.

### CFU enumeration.

Alongside each NAD(P)(H) extraction, an aliquot from the same culture was removed, serially diluted, and plated onto LB agar using a previously described single-plate serial dilution spotting method (101), and colonies were then grown overnight at 37°C.

### Western blotting.

The cultures were grown aerobically to an OD_600_ of 0.5 in MOPS minimal medium supplemented with the indicated carbon sources. The samples were centrifuged at 3,220 × *g* for 10 min. Equal amounts of protein were resolved on a 12% SDS-polyacrylamide gel. The proteins were blotted onto a nitrocellulose membrane, which was blocked with 5% (wt/vol) dried skimmed milk in Tris-buffered saline (TBS) buffer. The membranes were probed with mouse anti-NirS (102) and with IRDye 680RD and IRDye 800CW goat anti-mouse IgG secondary antibodies (Li-COR Biosciences; catalog no. 925-68070). Bands were visualized on an Odyssey infrared imaging system (Li-COR Biosciences).

### Fluorescence microscopy.

Fluorescence microscopy experiments were performed to determine bacterium length by the use of a custom-built microscope based on an IX-73 frame (Olympus, Center Valley, PA) with a 100× 1.49-numerical-aperture (NA) oil lens objective (Olympus UAPON100XOTIRF) and a 488-nm laser (Sapphire 488-300 CW CDRH [Center for Devices and Radiological Health]) (Coherent Inc.). Samples were imaged using epi-illumination microscopy, and the images were relayed onto an Andor iXon Ultra 897 camera by a ×1.3-magnification Cairn Twincam image splitter (the second port of the image splitter was not used during these experiments). The resulting pixel width on the sample was measured to be 118 nm. For each field of interest, 100 frames were captured at 100-ms exposure time in a 256-pixel^2^ region.

### Bacterial size analysis.

The micrographs captured for this study exhibited typically one or two bacteria in a 30-mm-by-30-mm area. The raw data were segmented and filtered for analysis using a MATLAB script (included in the supplemental material). The data were first segmented as follows. The MATLAB function adaptthresh was used to binarize the data using an adaptive threshold. This was deemed good enough for use as a segmentation step, since the bacteria were sparsely distributed over a dark background in a single frame. The MATLAB regionprops function was used to extract data representing the area, major axis length (length of the major axis of an ellipse fitted to the detected blob shape), eccentricity, and centroid of each segmented blob. A filtering step was implemented to include only those shapes detected with major axis lengths of between 1.7 and 4.5 μm (14 and 38 pixels), corresponding to the possible size ranges for the bacteria.

### Data availability.

The sequencing data were deposited at ArrayExpress (accession number E-MTAB-8374). The mass spectrometry proteomics data have been deposited in the ProteomeXchange Consortium via the PRIDE (54) partner repository with the data set identifier PXD015615.
